# GENE REGULATION IN AGING

**DOI:** 10.1093/geroni/igac059.1066

**Published:** 2022-12-20

**Authors:** Alexander Mendenhall

## Abstract

The regulation of genes can both influence aging, and be influenced by the aging process itself. That is, the rate of aging can be altered by changing the gene expression program. And, the aging process itself causes changes in gene expression, including the ways in which genes are regulated. Understanding the consequences of differences in gene expression on aging rate, and the consequences of aging on gene regulation will continue to have profound impacts on our ability to manipulate the aging process. This symposium on gene regulation in aging will focus on how genes are regulated by the aging process and can be regulated differently to affect the aging process. We have an expert on the regulation of gene expression in the immortal germline and soma of the hydra, Dr. Celina Juliano. Dr. Roger Brent is an expert on the mechanisms of cell to cell variation in gene expression. Dr. Monica Driscoll is an expert on both the genetics of aging and gene expression changes with age. Finally, Dr. Alex Mendenhall’s studies are focused on understanding intrinsic (epigenetic) variation in the regulation of gene expression as a cause and consequence of aging. Together these experts will present their research as it relates to gene regulation and the aging process.

